# Sex Differences in the Non-infarct-Related Artery-Based Quantitative Flow Ratio in Patients With ST-Elevation Myocardial Infarction: A Retrospective Study

**DOI:** 10.3389/fcvm.2021.726307

**Published:** 2021-09-24

**Authors:** Hongli Hou, Qi Zhao, Chao Qu, Meng Sun, Qi Liu, Xingtao Huang, Xuedong Wang, Ruoxi Zhang, Lifeng Du, Jingbo Hou, Bo Yu

**Affiliations:** ^1^Department of Cardiology, The 2nd Affiliated Hospital of Harbin Medical University, Harbin, China; ^2^Department of Cardiology, Chenjiaqiao Hospital of Chongqing Medical and Pharmaceutical College, Chongqing, China; ^3^Department of Cardiology, The 1st Affiliated Hospital of Harbin Medical University, Harbin, China; ^4^Department of Cardiology, Heilongjiang Provincial People's Hospital, Harbin, China; ^5^The Key Laboratory of Myocardial Ischemia, Harbin Medical University, Ministry of Education, Harbin, China; ^6^Department of Cardiology, Hegang People's Hospital, Hegang, China

**Keywords:** gender differences, non-infarct-related artery, ST-elevation myocardial infarction, quantitative flow ratio, revascularization

## Abstract

**Introduction:** It has been reported that sex has well-established relationships with the prevalence of coronary artery disease (CAD) and the major adverse cardiovascular events. Compared with men, the difference of coronary artery and myocardial characteristics in women has effects on anatomical and functional evaluations. Quantitative flow ratio (QFR) has been shown to be effective in assessing the hemodynamic relevance of lesions in stable coronary disease. However, its suitability in acute myocardial infarction patients is unknown. This study aimed to evaluate the sex differences in the non-infarct-related artery (NIRA)-based QFR in patients with ST-elevation myocardial infarction (STEMI).

**Methods:** In this study, 353 patients with STEMI who underwent angiographic cQFR assessment and interventional therapy were included. According to contrast-flow QFR (cQFR) standard operating procedures: reliable software was used to modeling the hyperemic flow velocity derived from coronary angiography in the absence of pharmacologically induced hyperemia. 353 patients were divided into two groups according to sex. A cQFR ≤0.80 was considered hemodynamically significant, whereas invasive coronary angiography (ICA) luminal stenosis ≥50% was considered obstructive. Demographics, clinical data, NIRA-related anatomy, and functional cQFR values were recorded. Clinical outcomes included the NIRA reclassification rate between men and women, according to the ICA and cQFR assessments.

**Results:** Women were older and had a higher body mass index (BMI) than men. The levels of diastolic blood pressure, troponin I, peak creatine kinase-MB, low-density lipoprotein cholesterol, N terminal pro B-type natriuretic peptide, stent diameter, and current smoking rate were found to be significantly lower in the female group than in the male group. Women had a lower likelihood of a positive cQFR ≤0.80 for the same degree of stenosis and a lower rate of NIRA revascularization. Independent predictors of positive cQFR included male sex and diameter stenosis (DS) >70%.

**Conclusions:** cQFR values differ between the sexes, as women have a higher cQFR value for the same degree of stenosis. The findings suggest that QFR variations by sex require specific interpretation, as these differences may affect therapeutic decision-making and clinical outcomes.

## Introduction

Coronary artery disease (CAD) is the leading cause of death globally, with the majority of affected individuals dying of acute myocardial infarction (1). Evidence from pathologic studies suggest that there are significant differences in coronary obstructive lesions between man and women (2, 3). Women are diagnosed with CAD at a greater age compared to men. In addition, women diagnosed with CAD have specific clinical symptoms, lower cardiovascular risk assessment scores, and a lower incidence of obstructive coronary heart disease, however, they appear to have a worse prognosis. Studies have shown lower utilization of conventional non-invasive imaging in female patients with suspected diagnosis of CAD, which may be related to breast attenuation, the decrease of peak exercise ability, small body size, and lower incidence of obstructive CAD (4). Therefore, sex-specific strategies and interpretations are recommended in the guidelines (5).

More recent evidence suggests that patients with non-infarct-related artery disease (NIRA) may in fact have a worse prognosis, including a higher 30-day mortality, than patients without NIRA disease (6). In addition, there is a lack of consensus regarding the treatment approach (aggressive vs. conservative) that should be implemented in these patients with NIRA disease. For some special research projects, patients with ST-elevation myocardial infarction (STEMI) underwent complete revascularization show lower rates of major cardiovascular events than infarct-related artery (IRA)-only revascularization (7, 8).

In recent years, fractional flow reserve (FFR) testing has become an important step in flow reconstruction decisions in patients with CAD, with the aim of assessing the functional significance of epicardial coronary stenosis on angiography.

Studies have shown that the choice of an FFR-based revascularization strategy is superior to percutaneous coronary intervention (PCI) for infarct-related vessels alone, both in STEMI procedure or in a staged manner (9, 10). However, the cost of the pressure wire and limitations associated with the induction of hyperemia have limited the wide application of FFR. A novel practical tool which calculates FFR indirectly and guides revascularization decision-making without a pressure wire or hyperemic induction was established. Recent studies have shown that the contrast-flow quantitative flow ratio (cQFR) can accurately and reproductively predict FFR values (11). Mechanically, the cQFR was calculated based on a contrast-flow hyperemic flow velocity model derived from coronary angiography without pharmacologically induced hyperemia. The accuracy of the cQFR in comparison with the conventional FFR has already been reported (12), and the software for the cQFR measurement is now commercially available.

Therefore, study aimed to evaluate the sex differences in the NIRA-based cQFR in patients with STEMI. We hypothesized that sex differences in NIRA might be related to the difference in revascularization decision-making based on the quantitative flow ratio (QFR) in patients with STEMI.

## Materials and Methods

### Ethics Statements

This study protocol conforms to the ethical guidelines of the 1975 Declaration of Helsinki, as reflected in a priori approval from the institution's human research committee. This study was also approved by the Research Ethics Committee of the 2nd Affiliated Hospital of Harbin Medical University, China. Informed consent was obtained from all patients.

### Study Population

We identified 353 patients admitted for STEMI who underwent angiographic QFR assessment and interventional therapy at the 2nd Affiliated Hospital of Harbin Medical University between January 2018 and December 2019. The inclusion criteria were as follows: (1) patients presenting within 12 h of symptom onset, defined as typical chest pain lasting for >30 min; (2) ST-segment elevation ≥1 mm in two contiguous electrocardiographic leads or new onset of complete left bundle branch block; and (3) primary PCI including balloon angioplasty, thrombus aspiration, and/or stent implantation. The exclusion criterion included any chronic illness, such as cancer, liver cirrhosis, heart failure, or end-stage renal failure (13).

### Coronary Angiography and Percutaneous Coronary Angiography

All primary PCIs were performed at our hospital [Department of Cardiology, The 2nd Affiliated Hospital of Harbin Medical University, a single tertiary interventional treatment center [>5,000 PCI cases per year]] by experienced interventional cardiologists, with experience with >1,000 PCI cases per year, who were not involved in the present study (13, 14). Baseline demographics and angiographic characteristics, as well as laboratory and physical examination data during hospitalization, were recorded by systematically reviewing the patients' files.

Primary PCI was usually performed using the percutaneous radial artery approach; the femoral approach was used when an intra-aortic balloon pump needed to be inserted. All patients' angiographic data from catheterization laboratory records were assessed using the conventional technique. The target artery was defined as clinically significant when vessel stenosis was ≥50%. Blood flow in the IRA that received only primary PCI was graded based on the thrombolysis in myocardial infarction (TIMI) grade. A chewable loading dose of 300 mg of aspirin and 600 mg of clopidogrel was administered before PCI. Success of the procedure was defined as <20% stenosis of the IRA with TIMI grade III flow after primary PCI. Post-operatively, all patients were transferred to our cardiac care unit and received standard treatment for STEMI, which consisted of 100 mg of aspirin, 20 mg of atorvastatin, and 75 mg of clopidogrel once a day (15).

### Figures Computation of the Contrast-Flow Quantitative Flow Ratio

**Figure 1:**
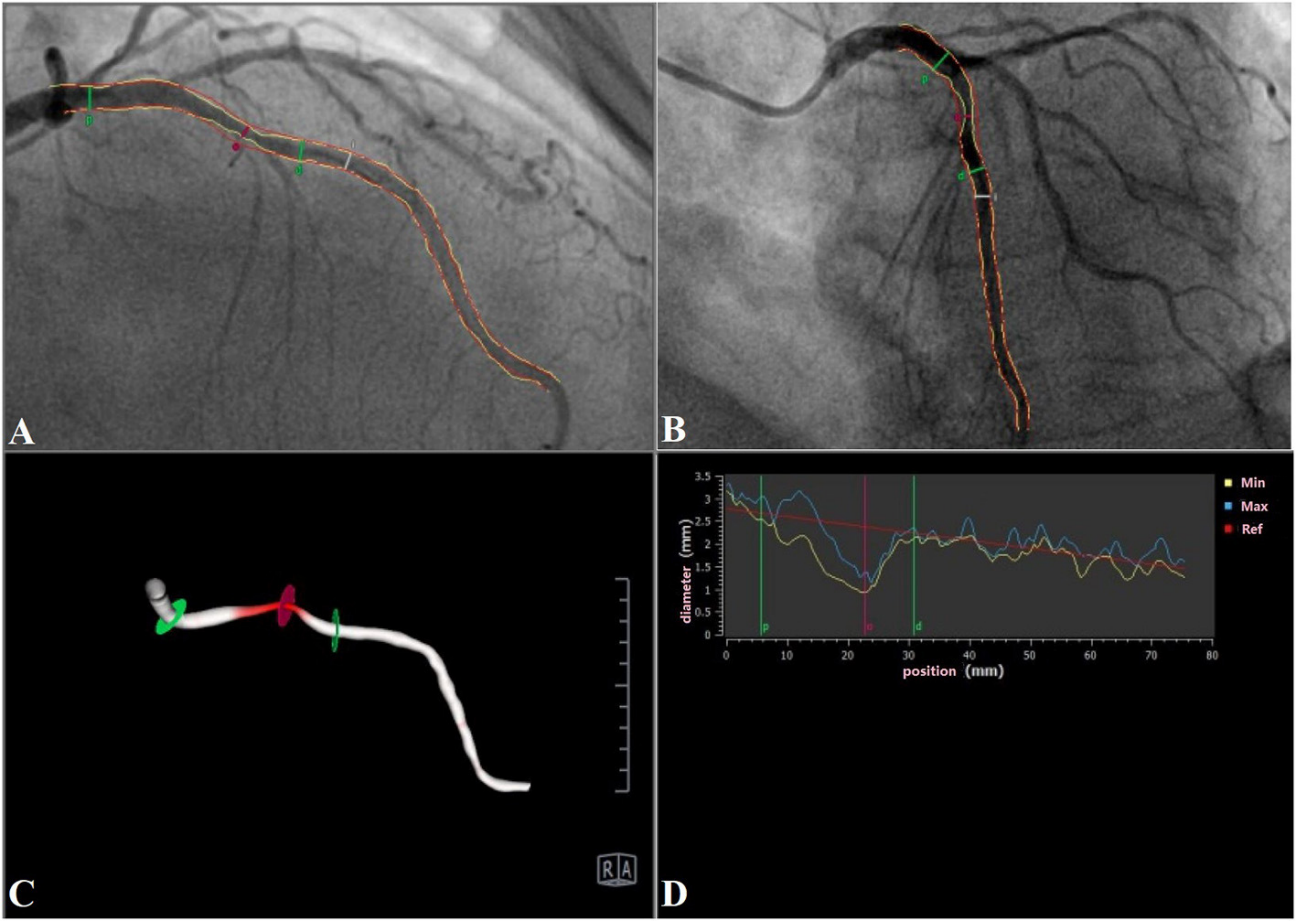
A representative case of LAD (cQFR = 0.71). **(A,B)** Two-dimensional images; **(C)** three-dimensional reconstruction (right anterior oblique 50°, caudal 9°); **(D)** diameter diagram. LAD, left anterior descending artery; cQFR, contrast-flow quantitative flow ratio.

Three-dimensional quantitative coronary angiography (QCA) and QFR analyses were performed by an independent core laboratory with dedicated software (QAngio XA 3D prototype, Medis special by, Leiden, the Netherlands) (Figures 1A–D). Briefly, end-diastolic frames of two optimal angiography projections, which were separated with angles of at least 25°, were selected and used for three-dimensional model reconstruction. Fifteen frames/second acquisition in our software that performs QFR was require. The three-dimensional contour model of the segment of interest and its reference vessel were constructed in an automated manner, and manual correction of the contour was performed. After acquisition of the fixed QFR, estimated contrast coronary flow was calculated using the TIMI frame-count adjustment, which indicated the frames where contrast entered and exited the segmented part of the vessel (12). With the application of the TIMI frame-count adjustment in the calculation method, the software automatically calculated the cQFR value. The cutoff value of the cQFR ≤0.80 was used in the current study (16).

### Blood Sampling

Venous blood was collected from all patients within 72 h of hospitalization. N terminal pro B-type natriuretic peptide (NT-proBNP), creatine kinase-MB (CK-MB), and troponin I levels were measured daily. Blood samples were collected on admission and at 24 and 48 h in every patient. Plasma NT-proBNP levels were measured using an Elecsys 2010 analyzer, a commercially available electrochemiluminescent sandwich immunoassay (Elecsys proBNP; Roche Diagnostics, Mannheim, Germany) (14). High-sensitivity-C-reactive protein levels were measured using a commercially available immunonephelometric kinetic assay (BN ProSpec; Siemens, Tarrytown, NY, USA). The 12-h fasting serum levels of triglycerides, total cholesterol, low-density lipoprotein cholesterol (LDL-C), and high-density lipoprotein cholesterol (HDL-C) were measured using standard methods (15). Other biochemical measurements were performed using the standard methods.

### Definitions

Reperfusion time was defined as the symptom-to-balloon time, and the door-to-balloon time was defined as the time between hospitalization and balloon dilation (14). Diabetes mellitus was diagnosed when a patient was on insulin or oral hypoglycemic drugs or in patients not on insulin or oral hypoglycemic drugs but with a casual plasma glucose level >11.1 mmol/L, fasting plasma glucose level >7 mmol/L, or glycated A1c hemoglobin level >6.5%. Hypertension was diagnosed when the systolic arterial pressure was >140 mmHg and/or diastolic arterial pressure was >90 mmHg, and when the patient had been on antihypertensive drugs for a long time. Hyperlipidemia was defined as a fasting total serum cholesterol level >5.17 mmol/L, LDL-C level >3.15 mmol/L, or serum triglyceride level >1.70 mmol/L, or if the patient was on lipid-lowering agents owing to a medical history of hypercholesterolemia (15). Smoking was defined as current regular use of cigarettes or if the patient had quit smoking within the previous year. Cardiac function deterioration was defined as New York Heart Association (NYHA) functional class leveled up in follow-up compared to in hospital.

### Statistical Analyses

Quantitative variables are expressed as the mean ± standard deviation, and qualitative variables are expressed as the total number and percentage. The independent two-sample *t*-test was used to assess the differences between multiple sets of data. Categorical variables were compared using the chi-square or Fisher exact tests. Independent predictors of NIRA revascularization were identified using multivariate logistic regression analysis. Statistical significance was defined as a two-sided *p*-value <0.05. All statistical analyses were performed using SPSS version 19.0 (IBM Corp., Armonk, NY, USA).

## Results

### Basic Characteristics

In our single-center study, we initially screened 515 STEMI patients who underwent angiographic QFR assessment between January 2018 and December 2019. One hundred and sixty-two patients with angiography were excluded because of limited quality of the images or inappropriate angle of projections. A total of 353 patients (224 men and 129 women) were enrolled in the study. Patients were categorized into two groups according to different sex. The baseline demographic and angiographic characteristics of the two groups are presented in Tables 1, 2, respectively. Compared to men, women were older and had a higher body mass index (BMI). The levels of diastolic blood pressure, troponin I, peak CK-MB, LDL cholesterol, and NT-proBNP, stent diameter, and current smoking rate were found to be significantly lower in the female group than in the male group.

**Table 1:** Patient demographics and clinical data.

	**Male group** **(***n*** = 224)**	**Female group** **(***n*** = 129)**	***P*** **-value**
Age, years	64.69 ± 5.67	68.28 ± 8.38	<0.001
BMI, kg/m^2^	26.02 ± 4.32	27.17 ± 2.93	0.003
Obesity, BMI ≥30 kg/m^2^	38 (16.96)	22 (17.05)	1.000
**Cardiovascular risk factors**			
Hypertension	141 (62.95)	88 (68.22)	0.355
Diabetes mellitus	40 (17.86)	26 (20.16)	0.671
Hyperlipidaemia	134 (59.82)	90 (69.77)	0.069
Current smoking	108 (48.21)	11 (8.53)	<0.001
Previous MI	27 (12.05)	9 (6.98)	0.147
Previous PCI	43 (19.20)	15 (11.63)	0.074
**Killip class on admission**			
I	170 (75.89)	94 (72.86)	0.528
II	33 (14.73)	23 (17.83)	0.453
III	13 (5.81)	7 (5.43)	1.000
IV	8 (3.57)	5 (3.88)	1.000
SBP, mmHg	123.05 ± 20.09	126.42 ± 34.30	0.309
DBP, mmHg	77.73 ± 12.30	73.33 ± 14.63	0.003
Platelet counts, × 10^9^/L	218.50 ± 83.78	218.78 ± 55.20	0.966
WBC, × 10^9^/L	10.89 ± 1.91	11.06 ± 5.53	0.732
Hemoglobin, g/L	134.20 ± 26.78	131.83 ± 20.73	0.354
Creatinine, μmol/L	85.44 ± 11.72	87.02 ± 27.37	0.533
Troponin I, μg/L	15.74 ± 3.50	14.14 ± 3.53	<0.001
Peak CK-MB, μg/L	35.29 ± 16.22	27.47 ± 11.15	<0.001
Total cholesterol, mol/L	4.50 ± 0.79	4.67 ± 1.43	0.240
LDL-cholesterol, mol/L	3.00 ± 1.15	2.74 ± 0.73	0.010
HDL-cholesterol, mol/L	1.18 ± 0.39	1.30 ± 0.47	0.008
Triglyceride, mol/L	2.24 ± 1.22	2.09 ± 0.84	0.173
hs-CRP	10.09 ± 3.31	10.21 ± 4.44	0.792
NT-proBNP, pg/mL	701.07 ± 75.56	666.29 ± 49.66	<0.001

*Data are presented as n (%) or mean ± SD. BMI, body mass index; MI, miocardial infarction; PCI, percutaneous coronary intervention. SBP, systolic blood pressure; DBP, diastolic blood pressure; WBC, white blood cell; CK-MB, creatine kinase-MB; HDL, high-density lipoprotein; hs-CRP, high-sensitivity C-reactive protein; LDL, low-density lipoprotein; NT-proBNP, N-terminal pro-brain natriuretic peptide*.

**Table 2:** Primary angiographic and interventional therapy characteristics.

	**Male group** **(***n*** = 224)**	**Female group** **(***n*** = 129)**	***P*** **-value**
Reperfusion time, min	280.41 ± 116.17	287.32 ± 167.82	0.679
Door-to-balloon time, min	69.19 ± 14.79	67.81 ± 15.95	0.412
**IRA, n (%)**			
LAD	109 (48.66)	71 (55.04)	0.270
LCX	42 (18.75)	32 (24.81)	0.221
RCA	73 (32.59)	26 (20.16)	0.014
Stents per patient	0.97 ± 0.23	0.95 ± 0.29	0.355
Stent length, mm	23.75 ± 7.84	23.52 ± 7.78	0.793
Stent diameter, mm	2.72 ± 0.69	2.43 ± 0.69	<0.001
IABP, *n* (%)	18 (8.04)	6 (4.65)	0.276
Femoral approach, *n* (%)	22 (9.83)	14 (10.85)	0.855

*Data are presented as n (%) or mean ± SD. IRA, infarct-related artery; LAD, center anterior descending coronary artery; LCX, center circumflex coronary artery; RCA, right coronary artery; IABP, intraaortic balloon pump*.

### Characteristics of the Quantitative Flow Ratio

**Figure 2:**
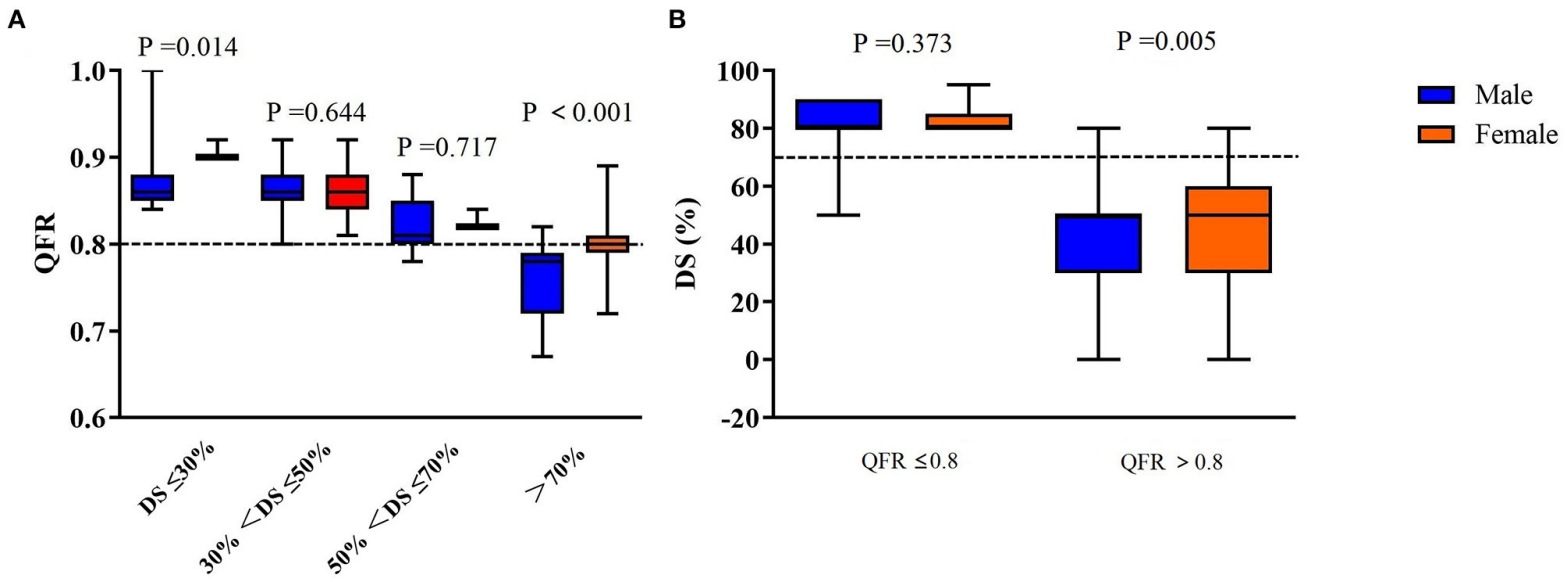
**(A)** cQFR values according to the severity of ICA stenosis. **(B)** Box-plot relationship of the anatomical degree of stenosis and cQFR values for men and women in the obstructive CAD categories. QFR, quantitative flow ratio; ICA, invasive coronary angiography; CAD, coronary artery disease; DS, diameter stenosis.

There was no significant statistical difference in each anatomical stenosis group (Table 3). Compared to men, women had a lower likelihood of a positive cQFR ≤0.80, a higher cQFR value in similar range of diameter stenosis (DS) (DS ≤30% and DS >70%), and a higher diameter stenosis while cQFR >0.80 (Figure 2). Multivariate logistic regression analysis showed that male sex [odds ratio [OR] = 2.240, 95% confidence interval [CI]: 1.237–3.941, *P* = 0.005], and DS >70% (OR = 9.407, 95% CI: 2.709–23.178, *P* = 0.001) were independently associated with positive cQFR (Table 4).

**Table 3:** Clinical characteristics on QCA and cQFR.

	**Male group** **(***n*** = 224)**	**Female group** **(***n*** = 129)**	***P*** **-value**
**Anatomical stenosis per patients**			
DS <30%	8 (3.57)	2 (1.55)	0.337
DS 30–50%	146 (65.18)	95 (73.64)	0.122
DS >70%	70 (31.25)	32 (24.81)	0.224
**Coronary vessel cQFR**			
LAD	0.84 ± 0.05	0.86 ± 0.04	0.003
LCX	0.83 ± 0.05	0.85 ± 0.05	0.018
RCA	0.82 ± 0.05	0.85 ± 0.04	<0.001
Overall	0.83 ± 0.05	0.86 ± 0.04	<0.001
cQFR≤0.8	82 (36.61)	22 (17.05)	<0.001
NIRA revascularization performed, *n* (%)	75 (33.48)	21 (16.30)	<0.001

*Data are presented as n (%) or mean ± SD. QCA, quantitative coronary angiography; cQFR, contrast-flow quantitative flow ratio; NIRA, non-infarct-related artery; LAD, center anterior descending coronary artery; LCX, center circumflex coronary artery; RCA, right coronary artery; DS, diameter stenosis*.

**Table 4:** Univariate and multivariate regression analysis for predicting positive cQFR.

	**Univariate**		**Multivariate**	
	**OR (95% CI)**	***P*** **-value**	**OR (95% CI)**	***P*** **-value**
Male	2.809 (1.648–4.787)	<0.001	2.240 (1.273–3.941)	0.005
DS >70%	9.694 (4.657-20.285)	<0.001	9.407 (2.709–23.178)	0.001
Previous MI	3.051 (1.515–6.142)	0.002		
Previous PCI	2.071 (1.160–3.700)	0.014		
Troponin I	1.088 (1.018–1.162)	0.012		
NT-proBNP	1.005 (1.001–1.008)	0.007		

*OR, odds ratio; CI, confidence interval; MI, miocardial infarction; PCI, percutaneous coronary intervention; DS, diameter stenosis*.

### One Year Follow-Up

**Figure 3:**
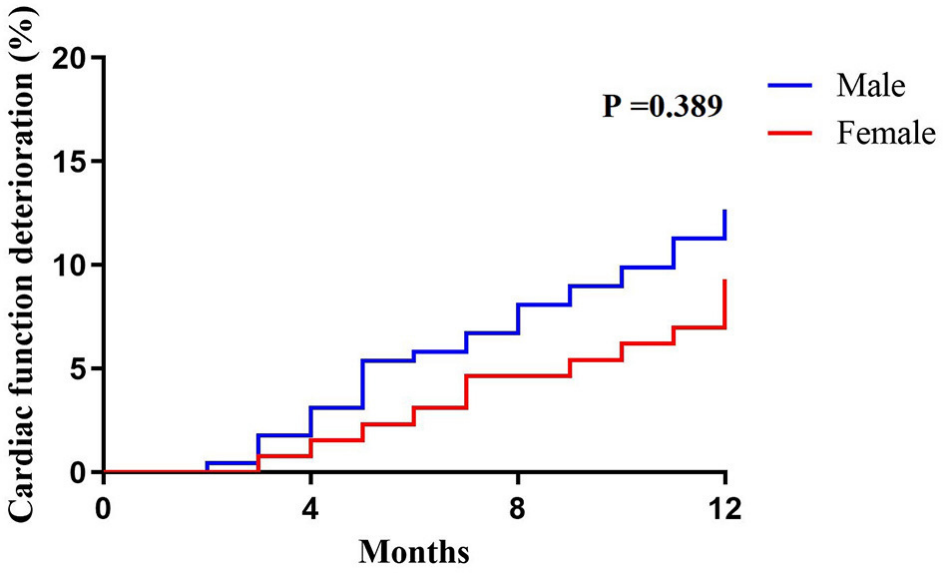
Cardiac function deterioration events between different sex in follow-up.

One-year follow-up the cardiac function deterioration events was trendly, but not significantly, higher in male group compared to female group (81.0 vs. 93.2%, *P* = 0.389; Figure 3).

## Discussion

QCA plays an important role in evaluating the degree of coronary artery stenosis and guiding the operation of PCI (17). However, limitations of anatomy-based techniques have required for a novel physiological technology to assess the severity of coronary stenosis (18, 19). The deep understanding of the anatomy and physiology of coronary circulation is necessary for clinical doctors to assess the hemodynamic consequences of epicardial stenosis and discover the personal clinical treatment strategy (20).

In fact, the adoption rates of invasive FFR-guided PCI remain low in real-world practice (21). In recent years, functional coronary imaging has been progressively applied, which is a new method based on mathematical assumptions of computational fluid dynamics models or coronary blood flow and thus assessing the degree of coronary artery stenosis (22). QFR is a three-dimensional QCA-based computation of FFR, moreover, previous studies have demonstrated excellent correlations and diagnostic agreements with FFR (23, 24). In addition, in some proof-of-concept studies, compared with FFR, QFR showed same excellent diagnostic ability whether in the acute physiological evaluation of non-culprit lesions in patients with STEMI or multivessel disease (25, 26).

Studies have shown that QFR showed good diagnostic agreement with FFR measured in the acute physiological evaluation of non-culprit lesions in patients with ST-segment elevation myocardial infarction (STEMI) and multivessel disease (26). To the best of our knowledge, this is the first time to investigate sex differences in NIRA-based QFR among patients with STEMI. Our results demonstrated several important sex-related differences. Compared to men, women have an inherently higher QFR value, independent of anatomical stenosis, and are less likely to receive revascularization of the NIRA. In the FAME substudy, it was found that the FFR value in women was 0.75 ± 0.18, whereas that in men was 0.71 ± 0.17 (*P* = 0.001). It is also reported that the proportion of functionally significant stenoses, defined as FFR <0.80, was lower in women than in men (27). For the patient cohort in our study, one-third of the patients were women. Compared to men, women were older, characteristic with higher BMI, and less cardiovascular risk factors (smoking, hypertension, and hypercholesterolemia). The above situation led to women might present with a lower number of coronary segments containing calcified or mixed plaques than men. Previous studies have highlighted sex differences in coronary stenoses, coronary plaque burden, and plaque composition, which may impact functional stenosis evaluation (28). For example, women presented with a lower number of coronary segments containing calcified or mixed plaques than men (29). In some quantitative and qualitative plaque characteristics studies, the number of high-risk plaque characteristics increased with decrease in FFR, and vice versa. Physiological disease burden (FFR) and the number of high-risk plaque characteristics were closely related, and both components had significant association with the risk of clinical events (28, 29). Furthermore, microvascular dysfunction is the most recognized reason for the higher FFR value in women, which is assessed by the coronary flow reserve, and it was reported to be more frequent in women than in men (30, 31). Accordingly, the blunted hyperemic response is considered as the major reason for the higher FFR value in women (20). Besides, compared with man, the smaller heart and myocardial perfusion territories are also important factors that cannot be ignored (32), which result in a relatively lower myocardial mass to coronary volume in women and a higher FFR value compared to men.

As expected in instances of disease severity, our results demonstrate that the average of QFR significantly higher in women, but importantly, the proportion of QFR-positive stenoses was also lower in women than in men (17.05 vs. 36.61%, *P* < 0.001). These results are highly consistent with those of the invasive Fractional Flow Reserve vs. Angiography for Multivessel Evaluation substudy (27). This difference in the sex-specific QFR/FFR offers new insights that may help to determine the appropriate treatment for women with CAD on interventional therapy. Our results emphasize that a high QFR value in women predicts a reduced likelihood of revascularization. Thus, our study confirms our hypothesis that sex differences in NIRA might be related to the difference in revascularization decision-making based on the QFR in patients with STEMI. Furthermore, because of women have a higher intrinsic FFR value, special consideration is required during PCI procedure: the strategies for revascularization need to be considered from multiple perspectives rather than using dichotomous cutoffs for invasive or non-invasive FFR (4). In our study, actual NIRA management for 90 days showed that women were more likely to receive medical treatment than men (83.70 vs. 66.52%, *P* < 0.001), and men were more likely to undergo NIRA revascularization than women (33.48 vs. 16.30%, *P* < 0.001). Therefore, the knowledge that women have a higher intrinsic physiological test value requiring special consideration, leading to suggestions that decisions of revascularization need to be nuanced and multifaceted rather than using dichotomous cutoffs for cQFR.

In previous studies, beyond sex and (as expected) % degree of stenosis, age, the presence of diabetes and vessel under study also showed a weak independent association with FFR data (33). In our study, female sex was significantly associated with positive cQFR after adjustment by DS (Table 4). In follow-up data, cardiac function deterioration rates were trendly, but not significantly, higher in male group compared to female group. There was also paradox that exists in female CAD, characterized by a lower prevalence of obstructive disease but higher prevalence of clinical presentation, ischemia, symptomatic complaints, complications and mortality compared to men (34). However, this difference in sex did not translate into a difference in cardiac function in our study. This might be due to recent advances in revascularization techniques, stent technology and medical therapies and the relatively low-risk population of this study.

## Study Limitations

The present study has some limitations. First, the number of patients was relatively small. There is a possibility of significant referral bias because of the retrospective and single-center design of the study. Second, we didn't measurement microvascular dysfunction, which leads to a lack of clarity understanding the differences in FFR values between men and women. Third, neither the physicians nor the patients were blinded to the QFR results and whether revascularization was performed. Finally, there is relatively insufficient in data on long-term events and follow-up, and we plan to include this information in a future study.

## Conclusions

There is significant different in QFR values between the male and female, as women have higher QFR values for the same degree of stenosis than men. Our findings suggest that QFR variations by sex needs more clinical and basic studies to explain the reasons, as these differences may affect clinical treatment strategy and long-term prognosis. However, the relative paucity of data supports the use of QFR at the NIRA in STEMI, and further investigation of the reliability of the non-culprit vessel QFR in this clinical setting is warranted.

## Data Availability Statement

The raw data supporting the conclusions of this article will be made available by the authors, without undue reservation.

## Ethics Statement

The studies involving human participants were reviewed and approved by The Research Ethics Committee of the 2nd Affiliated Hospital of Harbin Medical University. The patients/participants provided their written informed consent to participate in this study.

## Author Contributions

HH, RZ, JH, and BY were responsible for the conception and design of the study. HH, RZ, QZ, QL, XW, and XH contributed substantially to data acquisition. HH, QZ, CQ, QL, XW, LD, and XH contributed to data interpretation. MS was responsible for statistical analysis. HH and RZ drafted and revised the manuscript. All authors contributed to the article and approved the submitted version.

## Funding

This study was supported by the National Natural Science Foundation of China (grant nos. 81900309 to QL; 81801803 to XH; 82000330 to XW; and 8197020581 to JH), China Postdoctoral Science Foundation (grant nos. 2019M661306 to QL and 2018M640310 to XH), and Heilongjiang Postdoctoral Science Foundation (grant no. LBH-Z19032 to XH).

## Conflict of Interest

The authors declare that the research was conducted in the absence of any commercial or financial relationships that could be construed as a potential conflict of interest.

## Publisher's Note

All claims expressed in this article are solely those of the authors and do not necessarily represent those of their affiliated organizations, or those of the publisher, the editors and the reviewers. Any product that may be evaluated in this article, or claim that may be made by its manufacturer, is not guaranteed or endorsed by the publisher.
